# Arriving on time: decoding macrophage involvement in atrial fibrillation

**DOI:** 10.20517/jca.2023.28

**Published:** 2023-08-14

**Authors:** Yue Yuan, Na Li

**Affiliations:** 1 Cardiovascular Research Institute, Baylor College of Medicine, Houston, TX 77030, USA.; 2 Department of Medicine (Section of Cardiovascular Research), Baylor College of Medicine, Houston, TX 77030, USA.

Atrial fibrillation (AF) is the most common arrhythmia in adults, with a rising incidence and prevalence^[1]^. It is associated with increased risks of stroke, heart failure, and death. The risk of AF is associated with various factors, including aging, structural heart disease, obesity, hypertension, inflammation, and others. Rhythm control and rate control are the primary strategies for managing AF in clinical practice. While the focus of rate control is to slow down the heart rate without necessarily converting the abnormal rhythm to normal sinus rhythm, the more desirable rhythm control is to restore and maintain the normal sinus rhythm by utilizing cardioversion, antiarrhythmic drugs, or catheter ablation. The catheter-based ablation techniques have significantly progressed over the past several decades and generally yield satisfactory immediate results, yet the recurrence of AF remains a persistent challenge. Understanding the mechanisms that lead to AF is crucial for developing novel therapeutic strategies.

Historically, AF was primarily viewed as an electrophysiological disorder. However, AF is now increasingly recognized as an outcome of atrial myopathy, encompassing structural, functional, or electrical alterations within atria. The pathophysiology of AF typically integrates ectopic activity and the reentrant circuit-enabling substrate. The ectopic activity arises from augmented automaticity or cellular triggered activity due to the development of early or delayed afterdepolarizations, with the latter primarily resulting from aberrant Ca^2+^ release from the sarcoplasmic reticulum. AF substrate refers to either structural alterations like fibrosis or atrial enlargement, or functional alterations like an abbreviated effective refractory period or reduced conduction in atria. The development of substrate is a key for self-maintaining reentrant circuits; thus, it is recognized that the substrate is a root cause of recurrent AF.

Given the association of AF with numerous risk factors such as obesity and hypertension, exploring the causal link between these risk factors and the development of AF substrate may uncover key molecular mechanisms of AF with different etiologies. In recent years, growing evidence suggests that inflammation and immune cells are important players in many aspects of cardiac pathologies including AF^[2]^. The impact of inflammation and immune cell activation on atrial arrhythmogenesis could be several folds: first, the production and release of pro-inflammatory cytokines, such as interleukin (IL)-1b and IL-6, can modulate the electrophysiology and Ca^2+^ handling of cardiomyocytes via paracrine or autocrine mechanisms; second, cytokines can stimulate fibroblasts and promote collagen production, causing fibrotic remodeling and interrupted conduction; and third, limited evidence also suggests that immune cells could directly influence the action potential or electrophysiology of cardiomyocytes by forming direct contact^[3]^.

Recent advances in single-cell transcriptome analysis have revealed that myeloid and lymphoid cells are present in atria and can account for almost 10% of the atrial tissue of a healthy heart^[4]^. Notably, AF patients, even those without overt infections like myocarditis or sepsis, display pronounced monocyte infiltration in their left atria, which correlates with atrial enlargement^[5]^. This suggests a probable role for immune cells in structural remodeling that precipitates AF. In a recent publication in *Science*^[6]^, Hulsmans and colleagues have directly examined the causation between the recruited macrophages and the AF pathogenesis. In this study, by employing single-cell (sc)RNA-seq, they identified six major non-cardiomyocyte cell types in left atrial appendages from sinus rhythm control patients and persistent AF patients. Based on the descending order of relative cell numbers, these six cell clusters include - (1) lymphocytes; (2) mononuclear phagocytes and dendritic cells (MP/DCs); (3) endothelial cells; (4) fibroblasts; (5) mural cells; and (6) neutrophils. Notably, there was a twofold expansion in MP/DCs and a decline in endothelial and mural cells in AF patients. Gene set enrichment analysis (GSEA) revealed that the upregulated genes in MP/DCs are mostly related to the immunometabolic pathways. Using weighted correlation network analysis, the authors further identified 24 modules of correlated genes across all participants. Among three modules (Module 1, 2, 3) that are significantly correlated with AF state, Module 1 contains genes that are upregulated in AF patients and are known to be inflammatory and profibrotic, such as *CCR2*, *IL10*, *ITGA9, MMP9, SPP1, TIMP2*, and *VIM*. Intriguingly, additional clustering of MP/DCs revealed the *SPP1*^*+*^ subset of macrophages expanded more than 3-fold in the atria of AF patients. Although it is known that SPP1 encodes the matricellular protein osteopontin and is associated with fibrosis by promoting cell adhesion and migration, and inflammatory cell activation^[7]^, its involvement in AF pathogenesis has not been explored previously.

To further examine the role of macrophages in AF pathogenesis, the authors created a unique HOMER model by inducing hypertension (via angiotensin II infusion), obesity (via high-fat-diet feeding), and mitral valve regurgitation (via surgery) in mice, which mimics the three common risk factors of AF. Like AF patients, HOMER mice exhibited an enhanced susceptibility to the pacing-induced AF, accompanied by increases in left atrial volume, atrial fibrosis, and the numbers of macrophages and monocytes in atria. Importantly, scRNA-seq analysis with the left atria of HOMER mice largely mirrored the changes observed in the scRNA-seq dataset of AF patients. For instance, MP/DCs were expanded twofold in HOMER mice versus control mice, similar to that in AF patients with mitral regurgitation (MR). Spp1 was also markedly upregulated in HOMER mice, especially in two sub-clusters of macrophages that co-express *Trem2* and *Cd9*. Utilizing the genetic fate mapping approach, they revealed that the expanded atrial macrophages are mostly recruited and derived from monocytes in HOMER mice, suggesting the necessity of macrophage recruitment during atrial remodeling. These recruited macrophages are the primary origin of *Spp1* in HOMER atria. By inhibiting CCR2 signaling, which is crucial for macrophage recruitment, either through genetic deletion of *Ccr2* (*Ccr2*^*−/−*^) or using the CCR antagonist CCL2-Fc, the AF inducibility was lowered and left atrial volume was moderately reduced in the HOMER model. Since *Spp1* was the most upregulated gene in macrophages, they then went on to determine the role of SPP1 in AF development by introducing the bone marrow cells of *Spp1*^−/−^ mice into the wild-type mice that underwent the HOMER procedure. Deletion of *Spp1* in bone marrow cells - the source of myeloid cells not only reduced AF inducibility, but also lowered the CD68^+^ macrophage infiltration and collagen deposition in atria, compared with the HOMER mice received that wild-type bone marrow cells. Using the ligand-receptor repository^[8]^, the bioinformatic analysis revealed that SPP1^+^ macrophages in both AF patients and HOMER mice could engage with stromal cells, such as fibroblasts, via paracrine interactions and partake in the inflammatory fibroblast activation. The SPP1^+^ macrophages may release Spp1, which then binds to integrin receptors on fibroblasts, activating the transforming growth factor-β (TGF-β) pathway and promoting fibrotic remodeling [Figure 1].

This study presents several conceptual and technological innovations: First, the authors demonstrate direct evidence that the recruited macrophages contribute to the development of AF substrate, especially structural remodeling, such as atrial dilation and fibrosis. Second, the authors identified a subset of macrophages with high Spp1 expression that are activated in the combined condition of obesity, hypertension, and MR. Replicating the findings through scRNA-seq profiling in AF patients and the HOMER mouse model of AF strengthens the discovery of the Spp1 pathway. Third, the SPP1 signaling appears to be a key link between inflammation and fibrosis, facilitating atrial remodeling. Therefore, anti-macrophage therapies targeting the SPP1^+^ macrophages are showing potential in mitigating the adverse effects and limiting AF progression.

The establishment of the HOMER mouse model certainly recapitulates the characteristics of certain AF patients. However, it should be noted that not all AF patients exhibit these three risk factors simultaneously. Indeed, mouse models with only one or two of these conditions seem to lack the pronounced structural and electrophysiological changes observed in the HOMER model. In line with this observation, in a separate cohort of human atrial samples, the authors found that the CCR2^+^ macrophages expanded most in AF patients with MR. In contrast, this subset of macrophages expanded to a lesser degree in patients with AF alone or MR alone. Therefore, it remains undetermined whether the recruited macrophages play a role in other cohorts of AF patients with fewer or different risk factors. This group of investigators has previously demonstrated that Cx3cr1^+^ cardiac macrophages can electrically couple with cardiomyocytes via connexin 43, facilitating atrioventricular conduction. Ablating these macrophages can lead to atrioventricular block. However, this study did not directly assess whether and how the recruited macrophages influence the electrical remodeling in the atria. How CCR2-inhibition or Spp1-deletion affects atrial myocyte function in HOMER mice remains to be determined.

Nevertheless, Hulsmans and colleagues have once again highlighted the role of macrophages, especially the SPP1^+^ subset, in cardiac electrophysiology. Through mechanistic approaches, they demonstrated the involvement of these inflammatory cells in promoting a substrate for AF development. As a pleiotropic signal, SPP1 promotes AF via crosstalk between cardiac resident immune cells and non-immune cells. This study offers fresh insights into the emerging field of cardio-immunology.

## Figures and Tables

**Figure 1.**
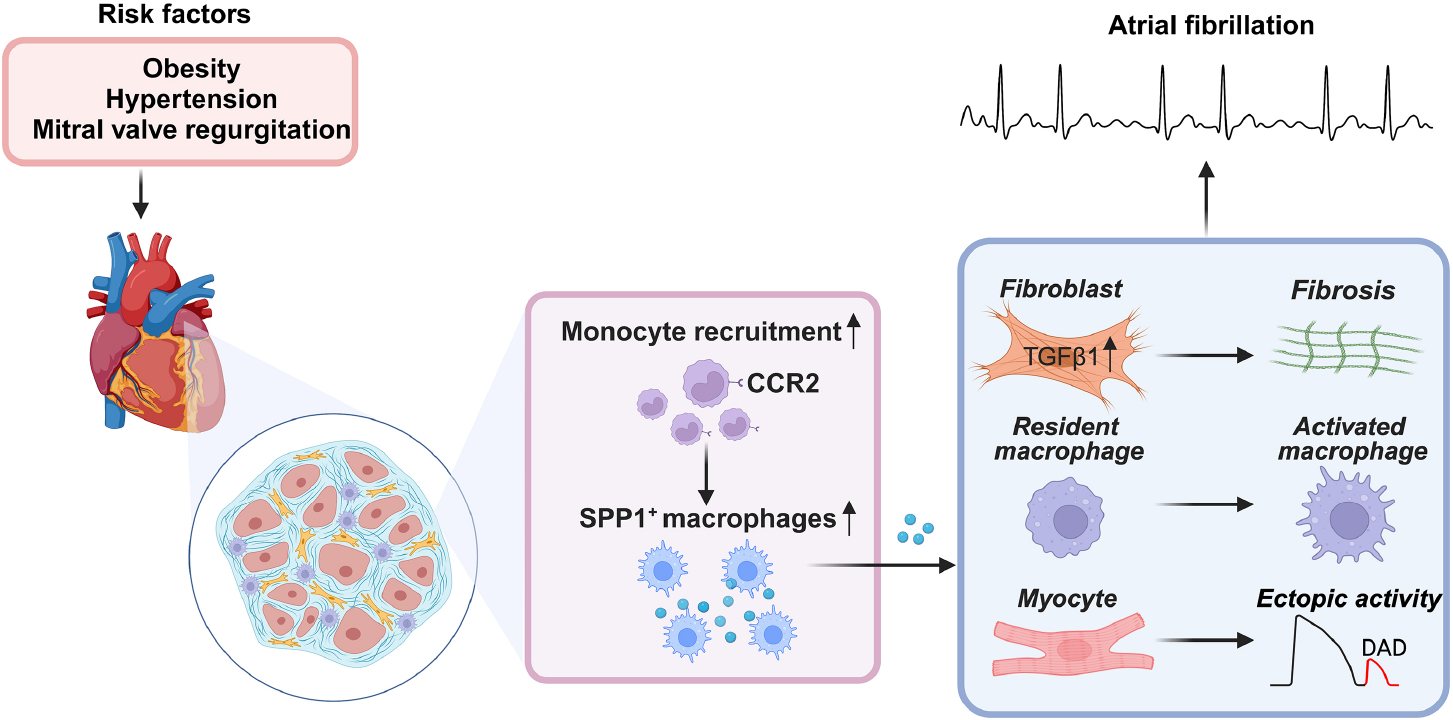
Working model: recruited SPP1^+^ macrophages promote the development of proarrhythmic substrate for atrial fibrillation. The figure is created with BioRender.com.

## Data Availability

Not applicable.
